# Effects of training and support programs for leaders of illness-based support groups: commentary and updated evidence

**DOI:** 10.1186/s13643-019-0981-0

**Published:** 2019-03-05

**Authors:** Kimberly A. Turner, Danielle B. Rice, Andrea Carboni-Jiménez, Jill Boruff, Brett D. Thombs

**Affiliations:** 1 Lady Davis Institute of the Jewish General Hospital, 4333 Cote Ste Catherine Road, Montréal, Québec H3T 1E4 Canada; 2 Department of Psychiatry, McGill University, Montreal, Quebec Canada; 3 Department of Psychology, McGill University, Montreal, Quebec Canada; 4 Schulich Library of Science and Engineering, McGill University, Montreal, Quebec Canada; 5 Department of Medicine, McGill University, Montreal, Quebec Canada; 6 Department of Educational and Counselling Psychology, Montreal, Quebec Canada; 7 Department of Epidemiology, Biostatistics, and Occupational Health, McGill University, Montreal, Quebec Canada

**Keywords:** Support groups, Leader training programs, Peer support, Peer leaders, Systematic review

## Abstract

**Background:**

Peer-led support groups play an important role in supporting people with chronic diseases. They may be particularly important for people with rare diseases who typically do not have access to professional support options that focus on their disease-specific needs. Many peer-led support groups in rare diseases, however, are not sustained, and many patients do not have access to support groups. Training and education for peer support group leaders could address barriers to initiating and sustaining groups, but there is little evidence on the effectiveness of support group leader training programs. A previous systematic review evaluated the effects of training programs for peer leaders of support groups for people with medical illness on leader and support group outcomes, but it identified only one randomized controlled trial (RCT) that compared high- and low-resource training programs for cancer support group leaders. The trial did not find evidence that the high-resource program was more effective, but was limited by a small sample size and serious methodological limitations. To meet the needs of people living with the rare autoimmune connective tissue disease scleroderma, the Scleroderma Patient-centered Intervention Network has partnered with patient organizations to develop the Scleroderma Support group Leader EDucation Program, and a full-scale RCT to test the effectiveness of the program is planned. To verify the need for such a trial, we updated the previous systematic review.

**Updated evidence:**

Review methods for the update were unchanged from the initial review. The updated database search yielded 1504 unique citations in addition to the 9757 assessed for eligibility in the previous review. All additional citations identified in the updated search were excluded at the title and abstract review stage.

**Conclusions:**

Our systematic review update found that there is presently insufficient evidence on the effectiveness of training and support programs for peer leaders of disease-based support groups, highlighting the need for well-designed and rigorously conducted RCTs to examine the effects of training for peer leaders of support groups, especially in a rare disease context. The Scleroderma Patient-centered Intervention Network’s trial of the Scleroderma Support group Leader EDucation Program will serve as such a trial.

**Systematic review registration:**

PROSPERO CRD42018096369

**Electronic supplementary material:**

The online version of this article (10.1186/s13643-019-0981-0) contains supplementary material, which is available to authorized users.

## Background

Illness-based support groups serve as an important source of support and education for many people with chronic medical illnesses [1–3]. Support group leaders have an integral role in determining the success and sustainability of these groups [4, 5]. Peer-led support groups often have an especially important role in supporting people with rare diseases, who typically do not have access to specialized support options through the health care system [6, 7].

Most peer leaders of support groups, however, do not receive any training. In a previous systematic review published in 2016 [8], we searched for evidence on whether training programs for peer leaders of support groups for people with medical illnesses (1) increase the competency and self-efficacy of leaders and (2) improve health outcomes and satisfaction with support groups among support group members. We searched for evidence through April 8, 2016, but identified only one randomized controlled trial (RCT) that met the inclusion criteria [9]. That trial compared the effects of a 2-day face-to-face group training workshop and 4-month access to a website and discussion forum (*N* = 29; high resource) to 4 months of website access and discussion forum alone (*N* = 23; low resource). The RCT did not find evidence that the high-resource program was more effective. However, the trial was substantially underpowered, not enough information was provided to determine the content of the intervention or how it was delivered, and risk of bias was rated as high due to methodological limitations [8].

### A training program for leaders of scleroderma support groups

Systemic sclerosis (SSc), or scleroderma, is a rare, chronic, autoimmune connective tissue disease characterized by abnormal fibrotic processes and excessive collagen production [10–12]. SSc is an example of a rare disease where peer-led support groups play an important role in helping people manage the impact of their disease [13–17]. Currently, there are between 150 and 200 active SSc support groups affiliated with Scleroderma Canada and the Scleroderma Foundation in the USA, most of which are led by people living with the disease [18, 19]. There are also support group networks in other countries. As is common in rare diseases, however, many people with SSc do not have access to SSc support groups, and many support groups that are initiated are not sustained due to a number of obstacles, some of which could be addressed by providing training to patient support group leaders [13–17].

To meet the needs of SSc patients, the Scleroderma Patient-centered Intervention Network (SPIN) [6, 20] partnered with SSc patient organizations to develop the Scleroderma Support group Leader EDucation (SPIN-SSLED) Program. The SPIN-SSLED Program is a 3-month long, 13-module, group training program that is delivered using videoconferencing in order to provide information and skills to improve patient support group leaders’ confidence and self-efficacy to carry out their leadership roles. Recently, SPIN undertook a feasibility trial of the program (NCT03508661), and a full-scale RCT is planned. To verify the need for such a trial, we updated our previous systematic review [8].

## Updated evidence

**Fig. 1:**
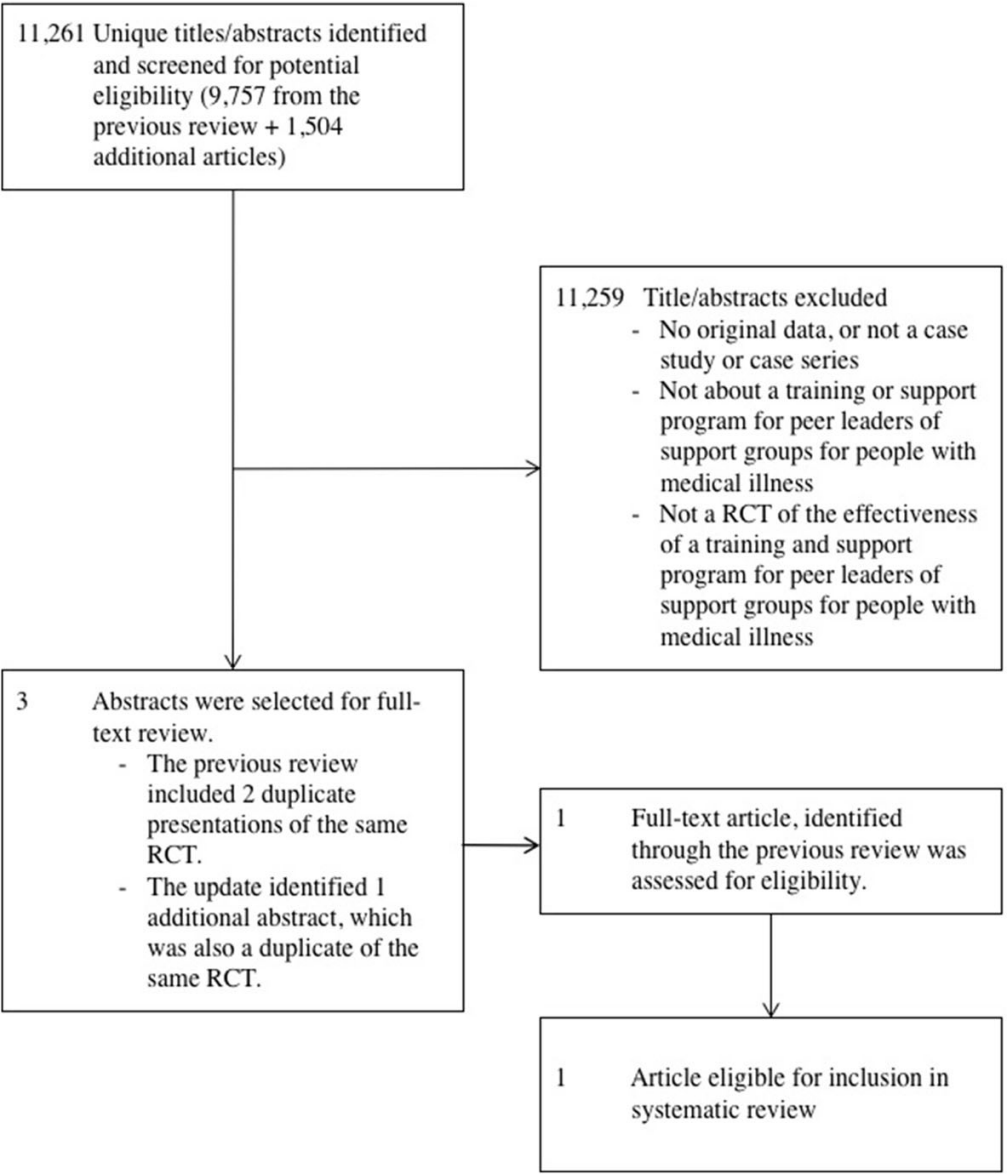
Updated PRISMA flowchart

The objective of the systematic review update was to seek updated evidence on effectiveness of training and support programs for peer leaders of support groups for people with medical illnesses on (1) the competency and self-efficacy of group leaders and (2) self-efficacy for disease management, health outcomes, and satisfaction with the support group experience among group members compared to inactive control groups and alternative training programs. A systematic review update was registered in PROSPERO (CRD42018096369). Review methods were unchanged from the initial review, which can be accessed via the open access publication (https://bmjopen.bmj.com/content/6/11/e013325.long) [8]. The updated search was done from the beginning of April 1, 2016, to June 4, 2018. The complete search strategy can be found in Additional file 1. The updated database search yielded 1504 unique article citations in addition to the 9757 assessed for eligibility in the previous review. See Fig. 1 for the updated PRISMA flowchart. All additional citations identified in the updated search were excluded at the title and abstract review stage. No additional eligible studies were identified via manual searching of the reference list of the included publication, by searching trial registries, or by citation tracking.

## Future directions

The original systematic review, which was published in 2016, did not identify any trials that compared peer support group leader training and support programs to no-training comparators. It identified one RCT that evaluated the effects of alternative training program resources [9]. That study did not report any statistically significant differences between the two training groups they evaluated, but only 65 support group leaders were randomized, the training programs provided were not well described, and there were risk of bias concerns across multiple domains [8]. The updated systematic review did not identify any additional evidence for inclusion.

There is a need for well-designed and rigorously conducted RCTs to examine the effects of training for peer leaders of support groups, especially in a rare disease context. Thus, SPIN has partnered with patient organizations, including Scleroderma Canada, the Scleroderma Foundation (USA), Scleroderma & Raynaud’s UK, and the Scleroderma Association of New South Wales (Australia) to develop and test the SPIN-SSLED Program, which is a videoconference-based training and education program, designed to improve skills and self-efficacy, reduce burden, and improve emotional and physical function among support group leaders. A Scleroderma Patient Advisory Team, comprised of current SSc peer support group leaders, and representatives from SSc patient organizations collaborated with a team consisting of researchers in SSc to develop the program. The program uses a learner-centered, problem-based learning approach that integrates theory and practice by first presenting relevant knowledge and skills, then presenting a real-world problem and working to identify solutions [21, 22]. The program consists of 13 modules delivered via weekly group videoconference sessions over the course of 3 months. Each session is approximately 60–90 min. A successful feasibility trial of the SPIN-SSLED Program was recently conducted (NCT03508661). Results informed revisions to the content of the program and provided confidence that the program can be effectively and efficiently delivered in a full-scale trial. The planned full-scale SPIN-SSLED trial, which was recently funded by the Canadian Institutes of Health Research, is scheduled to begin in 2019 (http://webapps.cihr-irsc.gc.ca/decisions/p/project_details.html?applId=388187&lang=en).

## Conclusions

In summary, peer-led support groups play an important role in supporting those who do not have access to professional support to provide educational and coping resources, which is often the case in rare diseases such as SSc. Our systematic review update found that there is presently insufficient evidence on the effectiveness of training and support programs for peer leaders of disease-based support groups. The SPIN-SSLED trial is intended to fill this knowledge gap by testing a program that is designed for support group leaders with the rare autoimmune disease scleroderma, but, if successful, will be easily adapted for other patient groups.

## Additional file

Additional file 1: Search strategies. (DOCX 14 kb)

## Abbreviations

PRISMA: Preferred Reporting Items for Systematic Reviews and Meta-analyses
PROSPERO: International prospective register of systematic reviews
RCT: Randomized controlled trial
SPIN: Scleroderma Patient-centered Intervention Network
SSc: Systemic sclerosis
SSLED: Scleroderma Support group Leader EDucation Program
USA: United States of America
