# Supplementary material for: Effects of training and support programs for leaders of illness-based support groups: commentary and updated evidence
Source: Syst Rev. 2019 Mar 5;8:67. doi: 10.1186/s13643-019-0981-0 (PMC6402093; doi:10.1186/s13643-019-0981-0)
Supplement: Supplementary file 1 — Search strategies. (DOCX 14 kb) [file 13643_2019_981_MOESM1_ESM.docx]

**ADDITIONAL FILE 1: Search Strategies**

**CENTRAL**

1.Self-Help Groups [MeSH descriptor (this term only)]

2. peer* support* or self help group or therapeutic social club* or peer* facilitat* or peer counseling [ti,ab,kw (word variations have been searched)]

3. 1 or 2

**CINAHL (via EBSCOhost)**

S1 (MH “Support Groups+”)

S2 (MH “Peer Counseling”)

S3 TX (therapeutic N1 social club*)

S4 TI ((non medical or non professional* or lay or layperson* or peer* or voluntary or patient) N1 (instructor* or tutor* or educator* or consultant* or leader* or expert* or advisor* or facilitat* or deliver* or mentor* or led or guide* or aide* or run))

S5 AB ((non medical or non professional* or lay or layperson* or peer* or voluntary or patient) N1 (instructor* or tutor* or educator* or consultant* or leader* or expert* or advisor* or facilitat* or deliver* or mentor* or led or guide* or aide* or run))

S6 TX randomized

S7 MH treatment outcomes

S8 PT clinical trial

S9 S6 OR S7 OR S8

S10 S1 OR S2 OR S3 OR S4 OR S5

S11 S9 AND S10

**EMBASE**

1. Self Help/

2. Group Support/

3. support* group*.tw.

4. group* support*.tw.

5. peer* support*.tw.

6. self help group*.tw.

7. (therapeutic adj social club*).tw.

8. ((non medical or non professional* or lay or layperson* or peer* or voluntary or patient) adj2 (instructor* or tutor* or educator* or consultant* or leader* or expert* or advisor* or facilitat* or deliver* or mentor* or led or guide* or aide* or run)).tw.

9. or/1-8

10. random*.tw.

11. clinical trial*.mp.

12. exp Health Care Quality/

13. exp Treatment Outcome/

14. double blind*.mp.

15. placebo*.tw.

16. blind*.tw.

17. or/10-16

18. exp animals/ not humans.sh.

19. 17 not 18

20. 9 and 19

**MEDLINE (via OvidSP)**

1. Self-Help Groups/

2. support* group*.tw.

3. group* support*.tw.

4. peer* support*.tw.

5. self help group*.tw.

6. (therapeutic adj social club*).tw.

7. ((non medical or non professional* or lay or layperson* or peer* or voluntary or patient) adj2 (instructor* or tutor* or educator* or consultant* or leader* or expert* or advisor* or facilitat* or deliver* or mentor* or led or guide* or aide* or run)).tw.

8. or/1-7

9. Randomized controlled trial.pt.

10. controlled clinical trial.pt.

11. randomi#ed.ab.

12. placebo.ab.

13. (clinical trials as topic or controlled clinical trials as topic or randomized controlled trials as topic).sh.

14. randomly.ab.

15. trial.ti.

16. or/9-15

17. exp animals/ not humans.sh.

18. 16 not 17

19. 8 and 18

**PsycINFO**

1. Support Groups/

2. Social Group Work/

3. peer* support*.tw.

4. Peer Counseling/

5. self help group*.tw.

6. (therapeutic adj social club*).tw.

7. ((non medical or non professional* or lay or layperson* or peer* or voluntary or patient) adj2 (instructor* or tutor* or educator* or consultant* or leader* or expert* or advisor* or facilitat* or deliver* or mentor* or led or guide* or aide* or run)).tw.

8. or/1-7

9. double-blind.tw.

10. random* assigned.tw.

11. control.tw.

12. or/9-11

13. exp animals/ not humans.sh.

14. 12 not 13

15. 8 and 14

**Web of Science (via ISI Web of Knowledge)**

1. TOPIC: (peer facilitat* or lay person facilitat* or layperson facilitat* or patient facilitat* or peer led or lay person led or layperson led)

2. TITLE: ((self help group* or support* group* or peer* support* or therapeutic social club* or social group work or peer counseling))

3. #2 AND #1
